# Interpreting Non-coding Genetic Variation in Multiple Sclerosis Genome-Wide Associated Regions

**DOI:** 10.3389/fgene.2018.00647

**Published:** 2018-12-17

**Authors:** Elvezia Maria Paraboschi, Giulia Cardamone, Giulia Soldà, Stefano Duga, Rosanna Asselta

**Affiliations:** ^1^Department of Biomedical Sciences, Humanitas University, Milan, Italy; ^2^Humanitas Clinical and Research Center, Milan, Italy

**Keywords:** multiple sclerosis, single-nucleotide polymorphism, association, long non-coding RNA, circular RNA, micro RNA, super-enhancer

## Abstract

Multiple sclerosis (MS) is the most common neurological disorder in young adults. Despite extensive studies, only a fraction of MS heritability has been explained, with association studies focusing primarily on protein-coding genes, essentially for the difficulty of interpreting non-coding features. However, non-coding RNAs (ncRNAs) and functional elements, such as super-enhancers (SE), are crucial regulators of many pathways and cellular mechanisms, and they have been implicated in a growing number of diseases. In this work, we searched for possible enrichments in non-coding elements at MS genome-wide associated loci, with the aim to highlight their possible involvement in the susceptibility to the disease. We first reconstructed the linkage disequilibrium (LD) structure of the Italian population using data of 727,478 single-nucleotide polymorphisms (SNPs) from 1,668 healthy individuals. The genomic coordinates of the obtained LD blocks were intersected with those of the top hits identified in previously published MS genome-wide association studies (GWAS). By a bootstrapping approach, we hence demonstrated a striking enrichment of non-coding elements, especially of circular RNAs (circRNAs) mapping in the 73 LD blocks harboring MS-associated SNPs. In particular, we found a total of 482 circRNAs (annotated in publicly available databases) vs. a mean of 194 ± 65 in the random sets of LD blocks, using 1,000 iterations. As a proof of concept of a possible functional relevance of this observation, we experimentally verified that the expression levels of a circRNA derived from an MS-associated locus, i.e., hsa_circ_0043813 from the *STAT3* gene, can be modulated by the three genotypes at the disease-associated SNP. Finally, by evaluating RNA-seq data of two cell lines, SH-SY5Y and Jurkat cells, representing tissues relevant for MS, we identified 18 (two novel) circRNAs derived from MS-associated genes. In conclusion, this work showed for the first time that MS-GWAS top hits map in LD blocks enriched in circRNAs, suggesting circRNAs as possible novel contributors to the disease pathogenesis.

## Introduction

Multiple sclerosis (MS) is a chronic autoimmune disease of the central nervous system, characterized by demyelination and progressive neurological impairment (Brownlee et al., 2017). Epidemiological studies showed both an important role of the environment in determining MS risk (Ramagopalan et al., 2010), and a strong contribution of genetic components (Belbasis et al., 2015). To date, besides the human leukocyte antigen (HLA) gene cluster (Patsopoulos et al., 2013), genome-wide association studies (GWAS) identified several common variants contributing to disease pathogenesis with mild effects on risk, many of which located within or close to genes displaying primarily immunologic functions (International Multiple Sclerosis Genetics Consortium [IMSGC] et al., 2007, 2011, 2013; Aulchenko et al., 2008; Comabella et al., 2008; Australia and New Zealand Multiple Sclerosis Genetics Consortium (ANZgene), 2009; Baranzini et al., 2009; de Jager et al., 2009; Jakkula et al., 2010; Nischwitz et al., 2010; Sanna et al., 2010; Patsopoulos et al., 2011; Martinelli-Boneschi et al., 2012; Matesanz et al., 2012). Despite these extensive efforts, the identified GWAS variants explain only 28% of the sibling recurrence risk (International Multiple Sclerosis Genetics Consortium [IMSGC] et al., 2013), thus implicating that the complete spectrum of MS genetic determinants is still far from being complete. These studies focused primarily on protein-coding genes, due to the difficulty of interpreting non-coding features. However, advances in the systematic annotation of non-coding genes and non-coding functional elements are revolutionizing genetic approaches and are paving the way to build a map that can help reveal “hidden” processes underlying disease associations (Ward and Kellis, 2012).

In this frame, non-coding RNAs (ncRNAs) have recently emerged as crucial regulators of many pathways and cellular mechanisms (Vidigal and Ventura, 2015; Barrett and Salzman, 2016; Quinn and Chang, 2016), and they have been implicated in a growing number of diseases (Mendell and Olson, 2012; Vučićević et al., 2014). Many long ncRNAs (lncRNAs), for instance, were shown to contribute to the pathogenesis of neurological and psychiatric conditions in different ways, from regulation of transcription to modulation of RNA processing and translation (Vučićević et al., 2014). In addition, microRNAs (miRNAs) dysregulation was associated with several disorders, such as different kinds of cancers and immune-related diseases (Mendell and Olson, 2012). Another group of ncRNAs with regulatory functions is represented by circular RNAs (circRNAs), a novel class of RNAs generated from the back-splicing of exons or introns (Jeck et al., 2013). By acting as miRNA sponges, or by binding to RNA-associated proteins, circRNAs regulate gene expression at the transcriptional or post-transcriptional level, although their exact mechanism of action still needs to be clarified (Greene et al., 2017). Moreover, they have been associated with human diseases such as ischemic heart disease, Alzheimer’s disease, diabetes, cancer, as well as MS (Cardamone et al., 2017; Greene et al., 2017; Iparraguirre et al., 2017).

Among non-coding functional elements, also super-enhancers (SEs) have been described as key gene expression regulators (Pott and Lieb, 2015). SEs are genomic regions characterized by a strong enrichment in binding sites both for transcriptional coactivators, specifically the Mediator protein, and for factors generally associated with enhancer activity, such as RNA polymerase II and chromatin factors (Pott and Lieb, 2015). Very interestingly, many SE regions are significantly enriched in disease-associated single-nucleotide polymorphisms (SNPs), including those related to autoimmunity, and more specifically to MS (Hnisz et al., 2013; Farh et al., 2015). The enrichment in GWAS variants within enhancers suggests that they influence the disease risk by altering gene regulation. However, only a few disease-associated SNPs directly alter a transcription factor motif; many trait-associated SNPs instead modulate the enhancer activity by changing nearby nucleotides, resulting in slight but critical alterations of gene expression (Farh et al., 2015).

In this work, we aim at identifying ncRNAs and SEs mapping in proximity of MS GWAS-significant signals that could point to so-far unexplored mechanisms involved in the susceptibility to the disease.

## Materials and Methods

### Defining the Linkage Disequilibrium (LD) Structure of the Italian Population

The global LD structure of the Italian population was explored by using genome-wide genotyping data (727,478 quality-checked markers, genotyped with the Affymetrix 6.0 GeneChip platform; Affymetrix, Santa Clara, CA, United States) obtained from 1,668 healthy controls (for genotyping details see Myocardial Infarction Genetics Consortium et al., 2009). Haplotype blocks were estimated with the Plink program (Purcell et al., 2007) following the default procedure described for the Haploview software (Barrett et al., 2005). Pairwise LD was calculated for SNPs within 200 kb for autosomal chromosomes. Chromosome X was excluded from this analysis, leading the total number of SNPs used for LD studies to 699,676.

To verify whether the Italian LD structure was comparable to the European one, we analyzed the 1000 Genomes data on European subjects (phase 1 project) (1000 Genomes Project Consortium et al., 2015). This test was performed on chromosome 22 data, by selecting only those SNPs whose genetic information was available both in the Italian and 1000 Genome populations. These were used to calculate the European LD structure.

### Retrieving the Reference Files for ncRNAs and Regulatory Elements

Reference files for the analysis were retrieved for lncRNAs, miRNAs, circRNAs, and SEs. In particular: (1) The reference gene transfer format (GTF) file for lncRNAs was obtained from GENCODE (Harrow et al., 2012), selecting the comprehensive gene annotation of lncRNA genes on the reference chromosomes, version 25^1^. (2) The miRNA reference file was downloaded from miRBase^2^ (Griffiths-Jones, 2004; Griffiths-Jones et al., 2006, 2008; Kozomara and Griffiths-Jones, 2011, 2014) version 20. (3) The circRNA reference file was obtained from the circBase database^3^ (Glažar et al., 2014), by downloading data from all the available studies on humans (Jeck et al., 2013; Memczak et al., 2013; Salzman et al., 2013; Zhang et al., 2013; Rybak-Wolf et al., 2015). (4) The SE reference file was downloaded from the SEA: Super-Enhancer Archive^4^ (Wei et al., 2016) based on studies on humans.

In all cases, genome version hg19 was considered; databases were accessed on April 2016.

### Defining Overlapping Regions Between LD Blocks, MS Genome-Wide Significant SNPs, and ncRNAs/SEs

Multiple sclerosis-associated SNPs, excluding those mapping in the highly complex HLA region, were retrieved from the literature (Supplementary Table 1) (International Multiple Sclerosis Genetics Consortium [IMSGC] et al., 2013). Their genomic coordinates were crossed with those of the LD blocks, to identify the blocks in which each single SNP resides.

The next step was searching for partial/total overlapping between LD blocks containing the genome-wide associated SNPs and the different classes of ncRNAs (lncRNAs, miRNAs and circRNAs) or SE elements. The overlaps were identified on the basis of the genomic coordinates of each LD block (using as borders the physical positions of the most 5′ and 3′ SNPs belonging to the block) and of each ncRNAs/SE elements (for these genomic features, coordinates were extracted from the reference files described in the previous section). The final list includes both the elements completely contained within the LD blocks and those showing only a partial overlap. Filtering for redundancy was used to eliminate multiple annotations referring to the same element. All procedures were performed using awk command line (described in the section Supplementary Material).

### Enrichment Analysis

To determine if the MS-related LD blocks are significantly enriched in ncRNA genes and SE elements, a bootstrapping strategy was adopted.

First, a set of random SNPs was extracted from the “Genome-Wide Human SNP array 6.0” manifest (copy number variants were excluded), which is one of the most used genotyping arrays in MS GWAS. The number of SNPs to be extracted was chosen in order to obtain either a number of LD blocks similar to the one of the MS-related analysis (Random set I) or an overall genomic region of equal length (i.e., 3.8 Mb; Random set II). Again, the HLA region and X chromosome were avoided. Moreover, since about half of the MS-associated SNPs are located in introns (Supplementary Table 1) (International Multiple Sclerosis Genetics Consortium [IMSGC] et al., 2013), the random SNP sets were constructed to mirror the proportion of intronic SNPs of the MS list. More in particular, to perform this step, two complete lists of SNPs from the “Genome-Wide Human SNP array 6.0” manifest were generated: one containing only SNPs annotated as intronic in the manifest file, the second containing only extragenic SNPs. SNPs were chosen from both lists with a randomized procedure (using the gshuf Unix command), respecting the constraints above mentioned.

Then, the LD blocks in which the random SNPs reside were identified, and a search for overlapping regions between LD blocks and ncRNAs/SE regions was performed, as described above.

Finally, the results were filtered to avoid redundancy, and the total number of lncRNAs, miRNAs, circRNAs, and SEs was annotated. The entire procedure was repeated 1,000 times for random set I and II, and the outputs of each set averaged, in order to compare the resulting means with the result obtained with the MS SNP set. The comparison was based on the % of times in which the same (or a larger) number of lncRNAs, circRNAs, miRNAs, or SEs was obtained in the 1,000 iterations respect to the MS dataset. Enrichment *p*-values were calculated according to Davison and Hinkley method (Davison and Hinkley, 1997).

All analyses were performed using in-house developed Perl scripts (listed in the section Supplementary Material).

The entire procedure is schematized in Supplementary Figure 1.

### Replication on an Unrelated Disease

To test the specificity of the analysis on MS, we repeated the entire workflow considering a disease with a completely different etiology, i.e., coronary artery disease (CAD).

The list of CAD-associated SNPs was derived from the literature (Supplementary Table 2) (Nikpay et al., 2015).

### Genotype-Dependent Analysis of circRNA Expression

DNA samples were extracted from whole blood of 35 healthy donors using an automated DNA extractor (Maxwell 16 System; Promega, Madison, WI, United States). All subjects gave written informed consent in accordance with the Declaration of Helsinki. To genotype the MS-associated SNP rs2293152, PCR amplifications (GoTaq; Promega) and Sanger sequencing, using the BigDye Terminator Cycle Sequencing Ready Reaction Kit v1.1 and an ABI-3500 Genetic Analyzer (Thermo Fisher Scientific, Waltham, MA, United States), were performed following standard protocols.

Peripheral blood mononuclear cells (PBMCs) of the same healthy donors were isolated by means of centrifugation on a Lympholyte Cell separation medium (Cederlane Laboratories Limited, Hornby, ON, Canada) gradient. RNA extraction was performed using the EuroGold Trifast kit (Euroclone, Wetherby, United Kingdom). RNA was reverse-transcribed using the Superscript-III Reverse Transcriptase (Thermo Fisher Scientific) and random hexamers (Promega), according to the manufacturers’ instructions.

Semi-quantitative real-time RT-PCRs to detect the expression levels of circRNA hsa_circ_0043813 were performed by using divergent primers (5′-ACATTCTGGGCACAAACACA-3′ and 5′-CCTCTGAGAGCTGCAACG-3′), the FastStart SYBR Green Master mix (Roche, Basel, Switzerland), and a LightCycler 480 (Roche). *HMBS* (hydroxymethylbilane synthase) was used as housekeeping gene; reactions were performed in triplicate, and expression data were analyzed using the GeNorm software (Vandesompele et al., 2002).

### CircRNA Analysis by RNA Sequencing

RNA was extracted using the Maxwell 16 LEV simplyRNA Cells Kit (Promega) from SH-SY5Y (human neuroblastoma) and Jurkat E6-1 (human T lymphocyte) cell lines. RNA quality was assessed by the LabChip GX Touch instrument (PerkinElmer, Waltham, MA, United States). RNA sequencing was performed using the TruSeq Stranded Total RNA Library Prep Kit (Illumina, San Diego, CA, United States), following the manufacturer’s instructions and a paired-end sequencing strategy. SH-SY5Y and Jurkat samples underwent a high-coverage paired-end 75- and 150-bp strand-specific sequencing, respectively, using a NextSeq 500 platform (Illumina).

The circRNA analysis was then performed using the DCC software (Cheng et al., 2016). In detail, raw reads were first aligned to the hg19 version of the genome using STAR (Dobin et al., 2013), switching on the detection of chimeric alignments to detect reads containing backspliced products, as suggested by the DCC manual. In a first step, reads were mapped using both mates; subsequently, an additional separate mate mapping was performed. After mapping, DCC was used to analyze the chimeric reads to detect circRNAs. Only those circRNAs supported by at least five reads were considered for further analyses. CircRNAs mapping on mitochondrial DNA or in repetitive regions of the genome were filtered out.

### Data Repository

Raw sequence files of SH-SY5Y have been deposited in NCBI Sequence Read Archive (SRA) under the following Bioproject ID: PRJNA483101, and with the accession number SRP155458; raw sequence files of Jurkat cells have been deposited in the GEO database (Accession No.: GSE110525).

## Results

### The Global LD Structure of the Italian Population Is Not Different From the European One

The LD structure of the Italian population was built by using data on 699,676 genotyped SNPs on 1,668 healthy subjects. A total of 96,666 LD blocks (with an average length of 17.48 kb; range 0.01–200 kb) were identified in autosomes, ranging from 1,421 blocks of chromosome 22 to 8,148 of chromosome 2 (on average: 4,394 blocks per chromosome).

The comparison of the LD structure of chromosome 22 of the Italian and European populations confirmed a substantial similarity in the blocks distribution (Pearson’s χ^2^ *p* = 0.79), thus suggesting that the Italian population is a good representation of the European LD structure, and confirming the data previously obtained by Mueller et al. (2005). The substantial overlap between the structures of the Italian and of the European populations was also confirmed by a principal component analysis performed using genotype data of our cohort and those from the 1000 Genome project (503 available individuals; Supplementary Figure 2).

### MS-Associated Regions Are Significantly Enriched in circRNAs and SEs

With the aim of identifying ncRNAs and SE regulatory elements mapping in LD blocks harboring MS GWAS-significant signals, we selected through literature data mining all those SNPs that reached a genome-wide significant threshold in MS GWAS studies and meta-analyses (Supplementary Table 1). We excluded from this list all SNPs mapping in the HLA region as well as those located on the X chromosome. The list of 97 SNPs was intersected with that of LD blocks inferred for the Italian population. Our automated pipeline allowed the identification of 73 LD blocks, each harboring a single genome-wide significant SNP. For 24 out of the 97 SNPs used for the analysis, it was not possible to establish a precise LD block.

The genomic coordinates of the 73 LD blocks were hence intersected with those of ncRNAs and SEs annotated in public genomic databases. This analysis evidenced the presence of 30 lncRNAs, 482 circRNAs, 7 miRNAs, and 23 SEs partially or totally overlapping the 73 identified blocks (Table 1). To test for possible significant enrichments in non-coding elements, the same workflow was applied to randomly selected SNPs (Random set I). To this aim, subsets of 94 SNPs were randomly selected from the genome, and the process was repeated 1,000 times. The pipeline identified, on average, 73.8 LD blocks (median value: 74) in which 22.6 lncRNAs (median: 22), 193.8 circRNAs (median: 185), 2.1 miRNAs (median: 2), and only 2.4 SEs (median: 2) were located (Table 1). Comparing the random set I results with the MS dataset, we obtained the same or a larger number of lncRNAs, circRNAs, and miRNAs in the 11, 0, and 3.6%, of the iterations, respectively, thus indicating a very strong enrichment in circRNAs in MS dataset. None of the randomly selected SNP subsets evidenced the same or a larger number of SE hits when compared to the MS list, thus confirming the enrichment in these regulatory elements previously reported by Farh et al. (2015) (observations that, however, were focused specifically on immune-related genes). Since the average length of the LD blocks in the Random set I was 22% lower than the one of MS LD blocks, we repeated the enrichment analysis on a genomic region spanning the same length as the MS dataset (3.8 Mb; Random set II). Also in this case, 1,000 iterations confirmed a strong enrichment in circRNAs and SEs in MS LD blocks (Table 1).

**Table 1:** Results of the analysis performed on the MS-associated loci (upper part) and on 1,000 random sets (middle and lower part).

List	*n* Blocks	Blocks§(kb)	DNA content (Mb)	*n* LncRNAs	*n* CircRNAs	*n* MiRNAs	*n* SEs
MS set	73	52.2	3.8	30	482	7	23
*SD*	–	54.8	–	–	–	–	–
Random sets (I)^∗^	73.8	40.6	2.9	22.6	193.8	2.1	2.4
*SD*	4.0	5.7	0.4	5.6	65.0	2.1	1.8
%^∗∗^	–	–	–	11.0	0	3.6	0
*p*-value	–	–	–	0.11	9.9^∗^10^-4^	0.36	9.9^∗^10^-4^
Random sets (II)^∗^	96.0	40.2	3.8	29.3	249.3	2.7	3.1
*SD*	4.6	4.8	0.5	6.3	76.4	2.5	2.0
%^∗∗^	–		–	46.4	0.6	6.3	0
*p*-value	–	–	–	0.46	0.006	0.06	9.9^∗^10^-4^

§*Average length of the LD blocks. ^∗^For the random sets analysis, the average values calculated on 1,000 iterations are indicated. ^∗∗^% of times in which the same or a larger number of lncRNAs, circRNAs, miRNAs, or SEs was obtained in the 1,000 iterations as compared to the MS dataset. p-Values were calculated as described (Davison and Hinkley, 1997); in detail: p-value = [1+sum(s > = s0)]/(N + 1), where s is the observed value in the random set, s0 is the value of the observed MS-specific result, and N is the number of bootstraps. *n*, number; SD, standard deviation.*

To test the specificity of the results, we applied the same pipeline on another dataset, composed of SNPs associated with CAD, a disease with a different etiology from MS. In this case, 55 SNPs were retrieved from the literature and used for the analysis, and 36 LD blocks were identified (Table 2). The workflow evidenced the presence of 19 lncRNAs, 122 circRNAs, 1 miRNA, and 2 SEs mapping within the corresponding blocks. When compared to the CAD dataset, the random bootstrapping strategy applied 1,000 times evidenced the same (or a larger) number of lncRNAs, circRNAs, miRNAs, and SEs in the 4.6, 12.4, 45.9, and 26.7% of the iterations, respectively (Table 2), thus suggesting only a slight enrichment in lncRNAs in the CAD dataset. No significant enrichment was evidenced in circRNAs and SEs.

**Table 2:** Results of the analysis performed on the CAD-associated loci (upper part) and on 1,000 random sets (lower part).

List	*n* Blocks	*n* LncRNAs	*n* CircRNAs	*n* MiRNAs	*n* SEs
CAD set	36	19	122	1	2
Random sets^∗^	35.3	11.3	73.4	1.0	1.0
*SD*	2.7	4.1	42.5	1.4	1.1
%^∗∗^	–	4.6	12.4	45.9	26.7
*p*-value	–	0.05	0.12	0.46	0.27

^∗^*For the random sets analysis, the average values calculated on 1,000 iterations are indicated. *n*, number; SD, standard deviation. ^∗∗^% of times in which the same or a larger number of lncRNAs, circRNAs, miRNAs, or SEs was obtained in the 1,000 iterations as compared to the CAD dataset. p-Values were calculated as described in the footnote of Table 1.*

The list of circRNAs/SE elements mapping within MS- or CAD-specific blocks is given in the Supplementary Table 3.

### SNP rs2293152 Genotype Influences *STAT3* hsa_circ_0043813 Expression Levels

**FIGURE 1:**
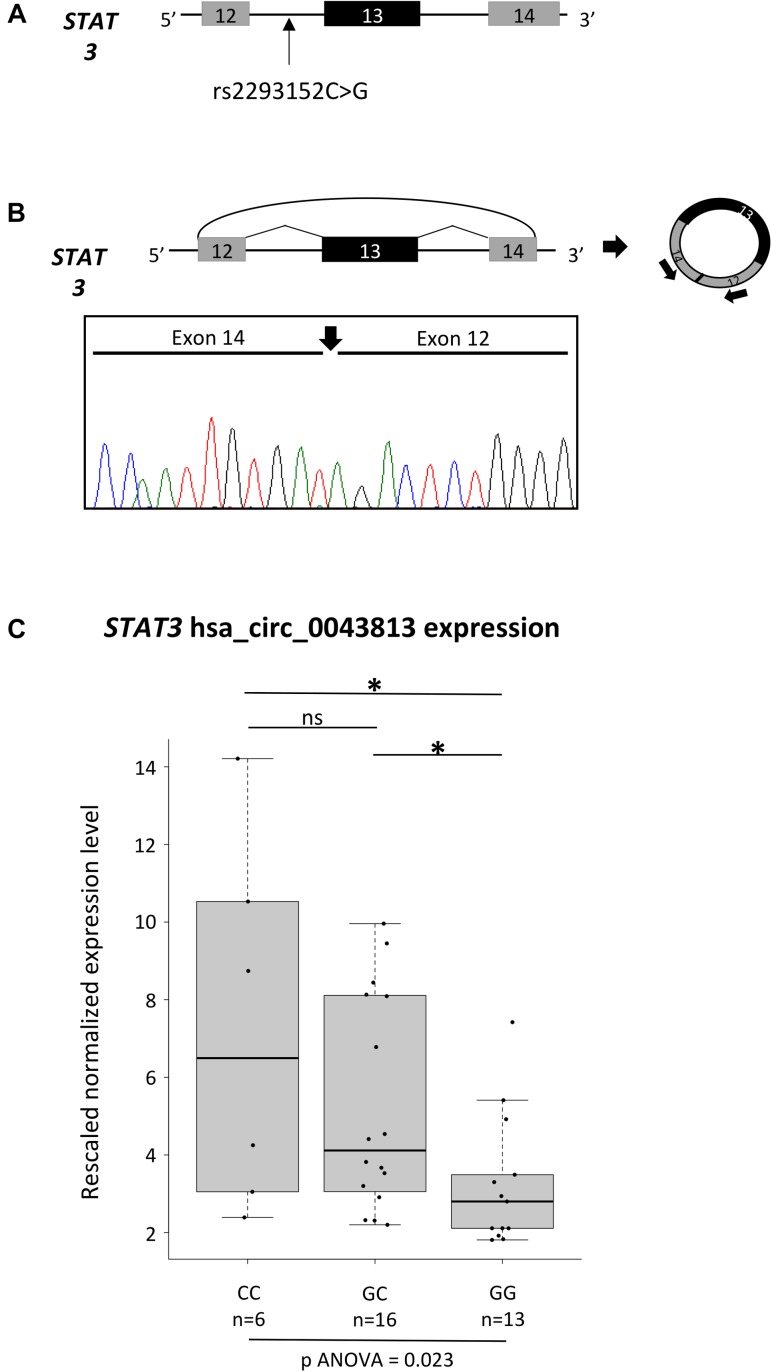
Characterization of the hsa_circ_0043813 circRNA deriving from the *STAT3* gene. **(A)** Schematic representation of the *STAT3* genomic region spanning from exon 12 to 14. Exons are depicted as boxes (in scale), and introns as lines. The position of the SNP rs2293152 is shown by an arrow. **(B)** Schematic representation of the formation of the *STAT3* circRNA hsa_circ_0043813 through a back-splicing event between exons 14 and 12. Exons are approximately drawn to scale; the curved arrow joins the 5′ splice site of exon 14 to 3′ splice site of exon 12. On the right, a schematic representation of the circRNA is depicted; arrows below exon 12 and 14 indicate the divergent primer couple used to detect the circRNA. Below the scheme, direct-sequencing electropherogram shows the head-to-tail splice junction, indicated by a black arrow, located between exons 14 and 12. **(C)** Boxplots showing expression levels of the hsa_circ_0043813 circRNA measured by semi-quantitative real-time RT-PCR in PBMCs of 35 healthy controls. Boxes define the interquartile range; the thick line refers to the median. Results were normalized to expression levels of the *HMBS* housekeeping gene, and for each sample three technical replicates were performed. The number of subjects belonging to each group is also indicated (n). The significance level of *t*-test analysis is shown. ^∗^*p* < 0.05; ns, not significant.

The newly observed enrichment in circRNAs in MS led us to test, as a proof of concept, whether the different genotypes of an MS-associated SNP could influence the expression levels of a circRNA mapping in the corresponding LD block. To this aim, we decided to better characterize a circRNA deriving from *STAT3* (Signal Transducer and Activator of Transcription 3), a gene necessary for pro-inflammatory cytokines signaling (Adamson et al., 2009) and that it is required for differentiation and expansion of Th17 cells, key players of MS disease activity (Brucklacher-Waldert et al., 2009). In particular, four different SNPs mapping in *STAT3* have been described as associated with MS in GWA studies: rs744166 (Jakkula et al., 2010), rs2293152 (Patsopoulos et al., 2011), rs9891119 (International Multiple Sclerosis Genetics Consortium [IMSGC] et al., 2011), and rs4796791 (International Multiple Sclerosis Genetics Consortium [IMSGC] et al., 2013). Among them, rs2293152 is in close proximity to a protein-coding exon, being located 50 nt upstream of *STAT3* exon 14 (Figure 1A), and could in theory affect the expression levels of the circRNAs hsa_circ_0043813 (CircBase, chr17:40481427-40481794, hg19), which is composed of exons 12, 13, and 14 (Refseq NM_003150).

To confirm the existence/expression of hsa_circ_0043813, we first performed a RT-PCR assay with a divergent primer couple tagging exons 12 and 13 on RNA extracted from PBMCs of two healthy controls. Direct sequencing of the circRNA product confirmed the presence of the backspliced exons 12 and 14, joined by a head-to-tail splice junction (Figure 1B). The analysis of the expression levels of the hsa_circ_0043813 circRNA was hence performed on a total of 35 healthy subjects: 6 homozygous for the CC genotype, 16 heterozygous, and 13 homozygous GG (Figure 1C). Our data showed significant different expression levels upon genotype stratification, with the CC subjects showing the highest levels of expression (one-way ANOVA *p* = 0.023).

### CircRNA Landscape in SH-SY5Y and Jurkat T Cell Lines

**FIGURE 2:**
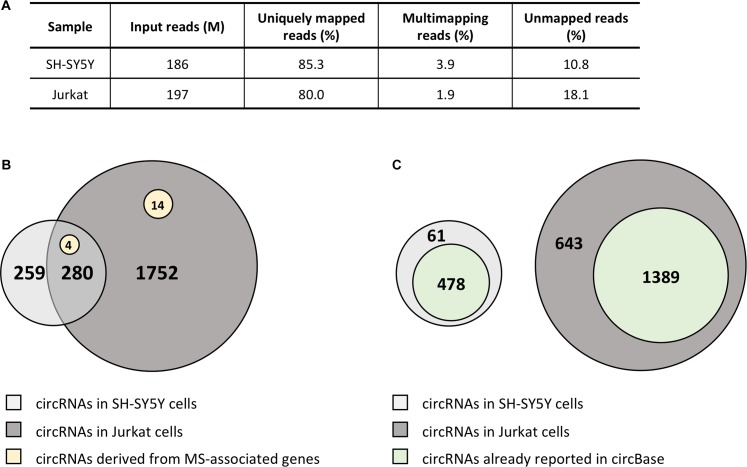
CircRNA landscape of SH-SY5Y and Jurkat cells. **(A)** Table showing alignment statistics of RNA-seq experiments in SH-SY5Y and Jurkat cells. M, million reads. **(B)** Venn diagrams representing the number of circRNAs shared by SH-SY5Y and Jurkat cells. The number of circRNAs identified only in one cell type is also shown. Yellow circles represent those circRNAs derived from MS-associated genes. **(C)** Venn diagrams representing the number of circRNAs that were already described in circBase for each cell line.

Due to the striking enrichment in circRNAs mapping in the regions associated with MS, we looked at the circRNA landscape of two cell lines, SH-SY5Y and Jurkat cells, representing tissues relevant for MS, by analyzing high-coverage RNA-seq data already available in our lab. We obtained ∼186 and 197 million reads for SH-SY5Y and Jurkat cells, respectively (Figure 2A). The circRNA analysis detected the presence of 539 circRNAs supported by at least five reads in SH-SY5Y cells, and of 2,032 circRNAs in Jurkat cells. About half (52%) of circRNAs identified in SH-SY5Y cells were also present in the Jurkat sample (Figure 2B). Most of the detected circRNAs were already annotated in circBase (89% for SH-SY5Y, 68% for Jurkat cells; Figure 2C). In Supplementary Table 3 we listed the 61 and 643 circRNAs that were newly identified in SH-SY5Y and Jurkat cells, respectively. Interestingly, we identified 18 (two novel) and 4 circRNAs derived from the MS-associated genes in Jurkat and SH-SY5Y cells, respectively (Figure 2B and Table 3).

**Table 3:** CircRNAs identified in Jurkat and SH-SY5Y cell lines within MS-associated genes.

Chr	Start^∗^	End^∗^	CircRNA ID^∗∗^	Gene	Jurkat	SH-SY5Y
3	119219542	119222868	hsa_circ_0001329	*TIMMDC1*	×	×
3	119219542	119232566	hsa_circ_0006884	*TIMMDC1*	×	
3	119219542	119236162	hsa_circ_0066874	*TIMMDC1*	×	
4	103610731	103651893	hsa_circ_0001431	*MANBA*	×	
4	103635595	103651893	hsa_circ_0142051	*MANBA*	×	
4	106155054	106158508	hsa_circ_0070562	*TET2*	×	×
7	50358644	50367353	hsa_circ_0001708	*IKZF1*	×	
7	50358644	50459561	novel	*IKZF1*	×	
7	50444231	50459561	novel	*IKZF1*	×	
16	11063018	11076848	hsa_circ_0004179	*CLEC16A*	×	
16	11114050	11145498	hsa_circ_0002086	*CLEC16A*	×	
16	11114050	11154879	hsa_circ_0000672	*CLEC16A*	×	
16	11114050	11220003	hsa_circ_0007846	*CLEC16A*	×	×
17	57808782	57816308	hsa_circ_0006508	*VMP1*	×	×
17	57808782	57851246	hsa_circ_0005077	*VMP1*	×	
22	22153301	22162135	hsa_circ_0004872	*MAPK1*	×	
22	22160139	22162135	hsa_circ_0008870	*MAPK1*	×	

^∗^*circRNA position is according to DCC output and referred to version hg19 of the genome. ^∗∗^circRNA identifier is referred to circBase nomenclature*.

Finally, *UBAP2* and *WHSC1*, and *MPP6* and *ZNF124* were the genes giving origin to the highest number of different circRNAs species in Jurkat and SH-SY5Y, respectively (Supplementary Figure 2). These genes show completely different genomic structures, going from 29 exons distributed on a region of 127 kb in the case of the *UBAP2* gene, to 4 exons spread over 16 kb for the *ZNF124* gene. Instead, for all these genes we observed highest level of expression in cell lines of lymphoid origin and in SH-SY5Y when compared to other cell lines (source^5^).

## Discussion

New classes of ncRNAs have been described over the last years; they all display regulatory functions, being part of a large RNA communication network that ultimately regulates the fundamental cellular functions (Adams et al., 2017). Many of them have in fact emerged as regulators of crucial mechanisms (Vidigal and Ventura, 2015; Barrett and Salzman, 2016; Quinn and Chang, 2016), and evidence suggests their implication in various diseases (Mendell and Olson, 2012; Vučićević et al., 2014). Given this background, in this work we aimed at identifying possible enrichments in non-coding elements at MS genome-wide associated loci, that could point to their involvement in the susceptibility to the disease.

By taking advantage of the top hits identified in MS GWASs and of the LD structure of the Italian population, we demonstrated a striking enrichment of circRNAs in the LD blocks harboring MS-associated SNPs. This result suggests that this class of ncRNAs could play an important role in the disease predisposition and supports emerging evidence in the literature indicating that a dysregulation of the back-splicing process could be a signature of the disease. More specifically, our group identified in MS patients, for the first time, one dysregulated circRNA (Cardamone et al., 2017) derived from *GSDMB*, a gene associated with susceptibility to asthma and autoimmune diseases. Subsequently, Iparraguirre et al. (2017) performed a microarray analysis identifying 406 differentially expressed circRNAs and validating two of them (both deriving from the *ANXA2* gene). As the biogenesis of circRNAs competes with pre-mRNA splicing (Ashwal-Fluss et al., 2014), alterations in the back-splicing process may also interfere with alternative splicing (AS), a mechanism already demonstrated to be dysregulated in MS (Evsyukova et al., 2010; Paraboschi et al., 2015).

Considering that AS dysregulation has been described as a possible pathogenic mechanism underlying autoimmune diseases (Evsyukova et al., 2010; Paraboschi et al., 2015; Juan-Mateu et al., 2016), and given the tight interconnection between AS and back-splicing, we hypothesized that an enrichment in circRNAs could be a signature also for other autoimmune disorders. Recent findings showed that immune-mediated diseases have a complex network of shared genetic architecture, with ∼70% of the associated loci for each disease being shared with other autoimmune disorders (Farh et al., 2015). We hence investigated whether we could identify a ncRNA signature also in systemic lupus erythematosus (SLE) and rheumatoid arthritis (RA), taking advantage of the GWAS top hits for these diseases (Okada et al., 2014; Bentham et al., 2015). By applying the same pipeline used for MS, we observed in SLE a circRNA enrichment in the LD blocks corresponding to GWAS signals (Supplementary Tables 3, 4). This result is in line with a growing body of evidence in the literature of an involvement of circRNAs in the disease: circHLA-C was in fact shown to be increased in patients affected by lupus nephritis (LN), a kidney disease caused by SLE. In addition, circHLA-C was correlated with clinical disease activities and was suggested to act as sponge for miR-150 (which, in turn, positively correlates with renal chronicity index in LN patients) (Luan et al., 2018). Regarding RA, we could not find any circRNA enrichment in the LD blocks corresponding to genome-wide associated loci (Supplementary Table 5). This finding, however, may not be so surprising: systematic reviews on familiar clustering of autoimmune disorders found evidence of an inverse clustering of RA and MS, suggesting that these two pathologies might be less closely related than other autoimmune diseases (Richard-Miceli and Criswell, 2012).

In our work, by studying *STAT3* hsa_circ_0043813, we also showed that the expression level of specific circRNAs may be influenced by the genotype of disease-associated SNPs (which might be defined as circ-eQTL). This observation could be very useful in understanding the functional impact of disease-associated SNPs, a task that still remains a key challenge of the post-GWAS era. Our hypothesis is that some variants associated with MS may impact on the biogenesis or on the sequence of circRNAs. This is in line with what has been reported for circANRIL, the only example of circRNA for which a link between disease associated SNPs and circRNA biogenesis has been demonstrated (Holdt et al., 2016). CircANRIL derives from the lncRNA ANRIL (Burd et al., 2010), transcribed from the CAD risk locus on chromosome 9p21. Holdt et al. (2016) demonstrated that carriers of the CAD-protective haplotype at this locus have significantly increased expression of circANRIL, and this is inversely correlated with the expression of linear ANRIL (linANRIL). Moreover, highest circANRIL:linANRIL ratios are found in CAD-free patients, thus implying an atheroprotective role of circANRIL. It is therefore likely that SNPs contained in the 9p21 haplotype are responsible for differential circANRIL formation, and that subtle genotype-directed gene expression differences may modulate the risk to develop the disease (Holdt et al., 2016). On the basis of this example, we can speculate that there might be other cases in which a disease-associated SNP exerts its functional effect by modulating the levels of specific circRNAs and, hence, modifying the ratio of the circular:linear isoforms. Of note, the RNA-seq analysis of the circRNA landscape in Jurkat cells highlighted the existence of 18 circRNAs deriving from seven MS-associated genes (∼8% of the total number of genes here considered; Supplementary Table 1). This group of circRNAs, together with their linear counterparts, could be a good starting point for an in-depth analysis of circular:linear isoform ratio in PBMCs of MS patients vs. controls, also in the perspective to find novel, simple, and reliable biomarkers for MS susceptibility and progression.

We are aware that our work has the potential limitation of comparing MS-associated loci, which are by definition non-random, with randomly sampled genomic regions. However, we think we have accounted for the main sources of bias by considering regions of equal length and exon density, and by performing a large number of iterations.

In conclusion, this work showed for the first time that MS-GWAS top hits map in LD blocks enriched in circRNAs, suggesting that this feature could be shared by other autoimmune diseases, and pointing to circRNAs as possible novel contributors to the disease pathogenesis.

## Ethics Statement

The study was approved by the Ethics Committee of the Humanitas Research Hospital and conducted according to the Declaration of Helsinki. All subject signed an appropriate informed consent.

## Author Contributions

EP and RA conceived and designed the experiments. GC and EP performed the experiments. RA and EP analyzed the data. EP drafted the paper. GC, GS, SD, and RA critically revised the manuscript. RA supervised the entire study.

## Conflict of Interest Statement

The authors declare that the research was conducted in the absence of any commercial or financial relationships that could be construed as a potential conflict of interest.

## Footnotes

1 https://www.gencodegenes.org/human/release_25lift37.html

2 http://www.mirbase.org/

3 http://www.circbase.org/

4 https://sea.nebulagene.com/SEA/index.html

5 https://www.proteinatlas.org/
