# Supplementary material for: Interpreting Non-coding Genetic Variation in Multiple Sclerosis Genome-Wide Associated Regions
Source: Front Genet. 2018 Dec 17;9:647. doi: 10.3389/fgene.2018.00647 (PMC6304422; doi:10.3389/fgene.2018.00647)
Supplement: Supplementary file 1 [file Data_Sheet_1.docx]

**SUPPLEMENTARY MATERIAL**

**Interpreting non-coding genetic variation in multiple sclerosis**

**genome-wide associated regions**

Elvezia Maria Paraboschi^1^, Giulia Cardamone^1^, Giulia Soldà^1,2^, Stefano Duga^1,2^, Rosanna Asselta^1,2*^

^1^Department of Biomedical Sciences, Humanitas University, Via Rita Levi Montalcini 4, 20090 Pieve Emanuele, Milan, Italy

^2^Humanitas Clinical and Research Center, Via Manzoni 56, 20089 Rozzano, Milan, Italy

**Content**

- - - - **Supplementary Methods.** Bioinformatic pipeline for enrichment analyses of disease-associated LD blocks.
- **Supplementary Table 1.** Susceptibility SNPs associated with MS
- **Supplementary Table 2.** Susceptibility SNPs associated with CAD
- **Supplementary Table 4.** Results of the analysis performed on the SLE-associated loci (upper part) and on 1,000 random sets (lower part)
- **Supplementary Table 5.** Results of the analysis performed on the RA-associated loci (upper part) and on 1,000 random sets (lower part)
- **Supplementary Figure 1.** Flowchart representing the pipeline steps.
- **Supplementary Figure 2.** Principal components of ancestry in Italians (our cohort) and Europeans (1000 Genome project subjects)
- **Supplementary Figure 3.** Distribution of circRNAs per gene
- **References**

**Note**

Supplementary Table 3 is contained in Excel file “Paraboschi et al - Supplementary table 3”

**SUPPLEMENTARY METHODS**

**Bioinformatic pipeline for enrichment analyses of disease-associated LD blocks.**

List of commands for the analysis of disease-associated LD blocks.

**#script1: create random subsets of SNPs extracted from the annotation file**

#!usr/bin/sh

for i in {1..1000}

do

gshuf -n xx list_SNP_bed_Intron > random_subset_$i

#xx: select the number of SNPs to extract

gshuf -n xx list_SNP_bed_nonIntron >> random_subset_$i

done

**#script2: for each random subset (or disease-specific list) find the blocks in which each SNP lies**

#!usr/bin/sh

for i in {1..1000}

do

perl block_finder.pl random_subset_$i random_subset_block_$i

done

#block_finder.pl

#!usr/bin/perl -w

use strict;

my @chr;

my @pos;

my @rs;

my $filename = $ARGV[0];

my $output = $ARGV[1];

#ARGV[0] is the list of SNPs (random_subset_$i); ARGV[1] is the output file (random_subset_block_$i)

open(SUBSET, "$filename") or die $!;

while (<SUBSET>) {

chomp;

my @col = split /\s/, $_;

push @chr, $col[0];

push @rs, $col[2];

push @pos, $col[1];

}

close SUBSET;

my @final;

open(OUT, ">$output") or die $!;

print OUT "chr(hg19)\tstart\tend\trs\tposizione_rs(hg19)\tSNPs\n";

#open the file containing the LD block structure

open(BLOCKS, "LD_structure.bed") or die $!;

while (<BLOCKS>) {

chomp;

my @col = split /\t/, $_;

for (my $i = 0; $i <= $#chr; $i++) {

if (("$col[0]" eq "$chr[$i]") and ($pos[$i] >= $col[1]) and ($pos[$i] <= $col[2])) {

push @final, "$chr[$i]\t$col[1]\t$col[2]\t$rs[$i]\t$pos[$i]\t$col[3]\n";

} else {}

}

}

close BLOCKS;

foreach (@final) {

print OUT;

}

**#script3: for each block identified with the script2, find the overlapping non-coding features**

#!usr/bin/sh

for i in $(seq 1 1000)

do

perl nc_blocks.pl random_subset_block_$i lnc_$i circ_$i mir_$i SE_$i

done

#nc_blocks.pl

#!usr/bin/perl -w

use strict;

my @chr;

my @start;

my @end;

my @finale;

my @circ;

my @mir;

my @sea;

my $filename = $ARGV[0];

my $output = $ARGV[1];

my $output2 = $ARGV[2];

my $output3 = $ARGV[3];

my $output4 = $ARGV[4];

#ARGV[0] is the output file from script2 (random_subset_block_$i), it contains the blocks in which SNPs lie; ARGV[1] is the output file produced by this script containing lncRNAs overlapping the blocks (lnc_$i); ARGV[2] is the output file produced by this script containing circRNAs overlapping the blocks (circ_$i); ARGV[3] is the output file produced by this script containing miRNAs overlapping the blocks (mir_$i); ARGV[4] is the output file produced by this script containing SEs overlapping the blocks (SE_$i)

#file LD blocks

open(BLOCKS, "$filename") or die $!;

while (<BLOCKS>) {

chomp;

my @col = split /\t/, $_;

push @chr, $col[0];

push @start, $col[1];

push @end, $col[2];

}

close BLOCKS;

open(GTF, "gunzip -c gencode.v25lift37.long_noncoding_RNAs.gtf.gz |") or die $!;

open(OUT, ">$output") or die $!;

open(OUT2, ">$output2") or die $!;

open(OUT3, ">$output3") or die $!;

open(OUT4, ">$output4") or die $!;

#file lncRNAs

while (<GTF>) {

chomp;

my @col = split /\t/, $_;

for (my $i = 0; $i <= $#chr; $i++) {

if ($_ =~ m/^##/){}

elsif (($col[2] eq "gene") and ($col[0] eq "$chr[$i]") and ($col[3] >= $start[$i]) and ($col[4] <= $end[$i])) {

push @finale, "$col[0]\t$col[2]\t$col[3]\t$col[4]\t$col[8]\n";

} elsif (($col[2] eq "gene") and ($col[0] eq "$chr[$i]") and ($col[3] <= $start[$i]) and ($col[4] >= $start[$i]) and ($col[4] <= $end[$i])) {

push @finale, "$col[0]\t$col[2]\t$col[3]\t$col[4]\t$col[8]\n";

} elsif (($col[2] eq "gene") and ($col[0] eq "$chr[$i]") and ($col[3] >= $start[$i]) and ($col[3] <= $end[$i]) and ($col[4] >= $end[$i])) {

push @finale, "$col[0]\t$col[2]\t$col[3]\t$col[4]\t$col[8]\n";

} elsif (($col[2] eq "gene") and ($col[0] eq "$chr[$i]") and ($col[3] <= $start[$i]) and ($col[4] >= $end[$i])) {

push @finale, "$col[0]\t$col[2]\t$col[3]\t$col[4]\t$col[8]\n";

} else {}

}

}

close GTF;

foreach (@finale) {

print OUT;

}

#file circRNAs

open(CIRC, "hsa_hg19_circRNA_human_download") or die $!;

while (<CIRC>) {

chomp;

my @col = split /\t/, $_;

for (my $i = 0; $i <= $#chr; $i++) {

if ($_ =~ m/^##/){}

elsif (($col[0] eq "$chr[$i]") and ($col[1] >= $start[$i]) and ($col[2] <= $end[$i])) {

push @circ, "$col[0]\t$col[1]\t$col[2]\t$col[4]\t$col[11]\n";

} elsif (($col[0] eq "$chr[$i]") and ($col[1] <= $start[$i]) and ($col[2] >= $start[$i]) and ($col[2] <= $end[$i])) {

push @circ, "$col[0]\t$col[1]\t$col[2]\t$col[4]\t$col[11]\n";

} elsif (($col[0] eq "$chr[$i]") and ($col[1] >= $start[$i]) and ($col[1] <= $end[$i]) and ($col[2] >= $end[$i])) {

push @circ, "$col[0]\t$col[1]\t$col[2]\t$col[4]\t$col[11]\n";

} elsif (($col[0] eq "$chr[$i]") and ($col[1] <= $start[$i]) and ($col[2] >= $end[$i])) {

push @circ, "$col[0]\t$col[1]\t$col[2]\t$col[4]\t$col[11]\n";

} else {}

}

}

close CIRC;

foreach (@circ) {

print OUT2;

}

#file miRNAs

open(MIR, "hsa_miRNA") or die $!;

while (<MIR>) {

chomp;

my @col = split /\t/, $_;

for (my $i = 0; $i <= $#chr; $i++) {

if ($_ =~ m/^#/){}

elsif (($col[0] eq "$chr[$i]") and ($col[3] >= $start[$i]) and ($col[4] <= $end[$i])) {

push @mir, "$col[0]\t$col[2]\t$col[3]\t$col[4]\t$col[8]\n";

} elsif (($col[0] eq "$chr[$i]") and ($col[3] <= $start[$i]) and ($col[4] >= $start[$i]) and ($col[4] <= $end[$i])) {

push @mir, "$col[0]\t$col[2]\t$col[3]\t$col[4]\t$col[8]\n";

} elsif (($col[0] eq "$chr[$i]") and ($col[3] >= $start[$i]) and ($col[3] <= $end[$i]) and ($col[4] >= $end[$i])) {

push @mir, "$col[0]\t$col[2]\t$col[3]\t$col[4]\t$col[8]\n";

} elsif (($col[0] eq "$chr[$i]") and ($col[3] <= $start[$i]) and ($col[4] >= $end[$i])) {

push @mir, "$col[0]\t$col[2]\t$col[3]\t$col[4]\t$col[8]\n";

} else {}

}

}

close MIR;

foreach (@mir) {

print OUT3;

}

#file SEs

open(SEA, "superenhancer.bed") or die $!;

while (<SEA>) {

chomp;

my @col = split /\t/, $_;

for (my $i = 0; $i <= $#chr; $i++) {

if ($_ =~ m/^#/){}

elsif (($col[1] eq "$chr[$i]") and ($col[2] >= $start[$i]) and ($col[3] <= $end[$i])) {

push @sea, "$col[1]\t$col[2]\t$col[3]\t$col[4]\t$col[5]\t$col[6]\n";

} elsif (($col[1] eq "$chr[$i]") and ($col[2] <= $start[$i]) and ($col[3] >= $start[$i]) and ($col[3] <= $end[$i])) {

push @sea, "$col[1]\t$col[2]\t$col[3]\t$col[4]\t$col[5]\t$col[6]\n";

} elsif (($col[1] eq "$chr[$i]") and ($col[2] >= $start[$i]) and ($col[2] <= $end[$i]) and ($col[3] >= $end[$i])) {

push @sea, "$col[1]\t$col[2]\t$col[3]\t$col[4]\t$col[5]\t$col[6]\n";

} elsif (($col[0] eq "$chr[$i]") and ($col[2] <= $start[$i]) and ($col[3] >= $end[$i])) {

push @sea, "$col[1]\t$col[2]\t$col[3]\t$col[4]\t$col[5]\t$col[6]\n";

} else {}

}

}

close SEA;

foreach (@sea) {

print OUT4;

}

**#script4: Compute the statistics on the files produced**

#!usr/bin/sh

for i in $(seq 1 1000)

do

awk -F'\t' '{print $5}' lnc_$i | awk -F';' '{print $1}' | sort | uniq | wc -l >> lnc_count

awk -F'\t' '{print $4}' circ_$i | sort | uniq | wc -l >> circ_count

grep -w miRNA mir_$i | awk -F'\t' '{print $5}' | awk -F';' '{print $3}' | sort | uniq | wc -l >> mir_count

wc -l block_$i >> blocks_count

awk -F'\t' '{print $1,$2,$3}' SE_$i | sort | uniq | wc -l >> SE_count

done

**Supplementary Table 1.** Susceptibility SNPs associated with MS.

| **Chromosome** | **SNP** | **Gene** | **p-value*** |
| --- | --- | --- | --- |
| 1 | rs3748817 | *MMEL1* | 1.3×10^−26^ |
| 1 | rs41286801 | *EVI5* | 1.4×10^−26^ |
| 1 | rs7552544 | *VCAM1* | 1.9×10^−16^ |
| 1 | rs6677309 | *CD58* | 5.4×10^−42^ |
| 1 | rs1359062 | *RGS1* | 4.8×10^−20^ |
| 1 | rs55838263 | *C1orf106* | 4.0×10^−19^ |
| 1 | rs3007421 | *PLEKHG5* | 4.7×10^−10^ |
| 1 | rs12087340 | *BCL10* | 1.1×10^−20^ |
| 1 | rs11587876 | *DDAH1* | 4.4×10^−9^ |
| 1 | rs666930 | *PHGDH* | 6.0×10^−12^ |
| 1 | rs2050568 | *FCRL1* | 1.5×10^−10^ |
| 1 | rs35967351 | *SLAMF7* | 4.4×10^−11^ |
| 2 | rs2163226 | Intergenic | 2.1×10^−16^ |
| 2 | rs7595717 | *PLEK* | 1.2×10^−13^ |
| 2 | rs9989735 | *SP140* | 4.2×10^−23^ |
| 2 | rs4665719 | *CENPO* | 3.1×10^−9^ |
| 2 | rs842639 | *FLJ16341* | 2.0×10^−14^ |
| 2 | rs9967792 | *STAT4* | 3.5×10^−12^ |
| 3 | rs2371108 | *EOMES* | 1.5×10^−15^ |
| 3 | rs1813375 | Intergenic | 1.9×10^−32^ |
| 3 | rs1131265 | *TIMMDC1* | 1.4×10^−23^ |
| 3 | rs1920296c | *IQCB1* | 6.5×10^−22^ |
| 3 | rs2255214c | *CD86* | 1.2×10^−24^ |
| 3 | rs1014486 | *IL12A* | 1.1×10^−18^ |
| 3 | rs11719975 | Intergenic | 1.1×10^−8^ |
| 3 | rs4679081 | *CCR4* | 2.2×10^−9^ |
| 3 | rs9828629 | *FOXP1* | 1.9×10^−10^ |
| 4 | rs7665090 | *MANBA* | 1.0×10^−8^ |
| 4 | rs2726518 | *TET2* | 3.9×10^−8^ |
| 5 | rs6881706 | *IL7R* | 4.3×10^−17^ |
| 5 | rs6880778 | Intergenic | 8.1×10^−20^ |
| 5 | rs71624119 | *ANKRD55* | 3.4×10^−13^ |
| 5 | rs756699 | *TCF7* | 8.8×10^−11^ |
| 5 | None | *NDFIP1* | 3.6×10^−9^ |
| 5 | rs4976646 | *RGS14* | 4.4×10^−18^ |
| 6 | rs72928038 | *BACH2* | 1.5×10^−15^ |
| 6 | rs11154801 | *AHI1* | 1.8×10^−20^ |
| 6 | rs17066096 | *IL22RA2* | 1.6×10^−23^ |
| 6 | rs67297943 | *TNFAIP3* | 5.5×10^−13^ |
| 6 | rs212405 | *TAGAP* | 8.0×10^−21^ |
| 6 | rs17119 | Intergenic | 1.0×10^−10^ |
| 6 | rs941816 | *PXT1* | 3.9×10^−12^ |
| 6 | rs7769192 | Intergenic | 3.3x10^-9^ |
| 7 | rs1843938 | *CARD11* | 1.2×10^−10^ |
| 7 | rs706015 | Intergenic | 1.1×10^−9^ |
| 7 | rs917116 | *JAZF1* | 3.3×10^−9^ |
| 7 | rs60600003 | *ELMO1* | 6.0×10^−14^ |
| 7 | rs201847125 | *IKZF1* | 1.2×10^−11^ |
| 8 | rs1021156 | *ZC2HC1A* | 8.5×10^−17^ |
| 8 | rs4410871 | *MIR1204* | 4.3×10^−16^ |
| 8 | rs759648 | *MIR1208* | 5.0×10^−10^ |
| 8 | rs2456449 | Intergenic | 1.8×10^−9^ |
| 10 | rs2104286 | *IL2RA* | 2.3×10^−47^ |
| 10 | rs1782645 | *ZMIZ1* | 2.5×10^−15^ |
| 10 | rs7923837 | *HHEX* | 4.3×10^−17^ |
| 10 | rs793108 | Intergenic | 6.1×10^−12^ |
| 10 | rs2688608 | *C10orf55* | 4.6×10^−8^ |
| 11 | rs34383631 | *CD6* | 3.7×10^−23^ |
| 11 | rs523604 | *CXCR5* | 6.2×10^−15^ |
| 11 | rs7120737 | *AGBL2* | 1.0×10^−9^ |
| 11 | rs694739 | *PRDX5* | 2.0×10^−9^ |
| 11 | rs9736016 | *CXCR5* | 3.0×10^−15^ |
| 11 | rs533646 | *TREH* | 7.6x10^-11^ |
| 12 | rs1800693 | *TNFRSF1A* | 6.7×10^−28^ |
| 12 | rs11052877 | *CD69* | 5.6×10^−13^ |
| 12 | rs201202118 | *TSFM* | 9.0×10^−22^ |
| 12 | rs7132277 | *PITPNM2* | 1.9×10^-13^ |
| 12 | rs12296430 | *LTBR* | 7.2×10^−14^ |
| 13 | rs4772201 | *MIR548AN* | 1.3×10^−10^ |
| 14 | rs2236262 | *ZFP36L1* | 2.5×10^−12^ |
| 14 | rs74796499 | *GALC* | 2.4×10^−20^ |
| 14 | rs12148050 | *TRAF3* | 5.1×10^−13^ |
| 15 | rs59772922 | *CTSH* | 1.2×10^−8^ |
| 15 | rs8042861 | *IQGAP1* | 2.2×10^−9^ |
| 16 | rs12927355 | *CLEC16A* | 6.4×10^−46^ |
| 16 | rs35929052 | *IRF8* | 5.9×10^−12^ |
| 16 | rs6498184 | *RMI2* | 7.4×10^−18^ |
| 16 | rs7204270 | *MAPK3* | 1.6×10^−11^ |
| 16 | rs1886700 | *CDH3* | 1.3×10^−8^ |
| 16 | rs12149527 | *WWOX* | 3.3×10^−11^ |
| 16 | rs7196953 | *MAF* | 1.0×10^−10^ |
| 16 | rs4780346 | *CLEC16A* | 4.4x10^-10^ |
| 17 | rs4796791 | *STAT3* | 3.7×10^−20^ |
| 17 | rs8070345 | *VMP1* | 2.2×10^−23^ |
| 17 | rs12946510 | *IKZF3* | 2.9×10^−9^ |
| 17 | rs4794058 | *NPEPPS* | 1.0×10^−13^ |
| 19 | rs1077667 | *TNFSF14* | 1.7×10^−24^ |
| 19 | rs34536443 | *TYK2* | 1.8×10^−14^ |
| 19 | rs11554159 | *IFI30* | 1.9×10^−24^ |
| 19 | rs8107548 | *DKKL1* | 5.7×10^−15^ |
| 19 | rs2288904 | *SLC44A2* | 1.6×10^−11^ |
| 19 | rs1870071 | *EPS15L1* | 2.0×10^−15^ |
| 20 | rs4810485 | *CD40* | 7.7×10^−16^ |
| 20 | rs2248359 | *CYP24A1* | 2.0×10^−13^ |
| 20 | rs17785991 | *SLC9A8* | 4.2×10^−8^ |
| 20 | rs2256814 | *SLC2A4RG* | 3.5×10^−9^ |
| 22 | rs2283792 | *MAPK1* | 5.5×10^−16^ |

Adapted from IMSGC et al. (2013)

* p-value is derived from the meta-analysis of discovery and replication phase.

**Supplementary Table 2.** Susceptibility SNPs associated with CAD.

| **Chromosome** | **SNP** | **Gene(s)** | **p-value*** |
| --- | --- | --- | --- |
| 1 | rs11206510 | *PCSK9* | 2.34x10^-08^ |
| 1 | rs17114036 | *PPAP2B* | 2.22x10^-13^ |
| 1 | rs646776 | *SORT1* | 9.01x10^-19^ |
| 1 | rs4845625 | *IL6R* | 3.93x10^-08^ |
| 1 | rs17464857 | *MIA3* | 4.18x10^-05^ |
| 1 | rs17465637 | *MIA3* | 3.52x10^-12^ |
| 2 | rs16986953 | *AK097927* | 1.45x10^-08^ |
| 2 | rs515135 | *APOB* | 3.09x10^-08^ |
| 2 | rs6544713 | *ABCG5-ABCG8* | 8.88x10^-07^ |
| 2 | rs1561198 | *VAMP5-VAMP8-GGCX* | 6.37x10^-10^ |
| 2 | rs2252641 | *ZEB2-ACO74093.1* | 5.16x10^-04^ |
| 2 | rs6725887 | *WDR12* | 9.51x10^-18^ |
| 3 | rs9818870 | *MRAS* | 2.21x10^-06^ |
| 4 | rs1878406 | *EDNRA* | 1.24x10^-06^ |
| 4 | rs7692387 | *GUCY1A3* | 7.35x10^-09^ |
| 5 | rs273909 | *SLC22A4-SLC22A5* | 1.24x10^-04^ |
| 6 | rs6903956 | *ADTRP-C6orf105* | 4.87×10^−12^ |
| 6 | rs12526453 | *PHACTR1* | 2.14x10^-20^ |
| 6 | rs17609940 | *ANKS1A* | 0.03 |
| 6 | rs10947789 | *KCNK5* | 1.63x10^-06^ |
| 6 | rs12190287 | *TCF21* | 1.07x10^-03^ |
| 6 | rs2048327 | *SLC22A3-LPAL2-LPA* | 2.46x10^-09^ |
| 6 | rs3798220 | *SLC22A3-LPAL2-LPA* | 4.66x10^-09^ |
| 6 | rs4252120 | *PLG* | 3.32x10^-03^ |
| 7 | rs2023938 | *HDAC9* | 1.36x10^-04^ |
| 7 | rs10953541 | *7q22* | 1.02x10^-05^ |
| 7 | rs11556924 | *ZC3HC1* | 5.34x10^-11^ |
| 8 | rs264 | *LPL* | 1.06x10^-05^ |
| 8 | rs2954029 | *TRIB1* | 2.61x10^-06^ |
| 9 | rs3217992 | *9p21* | 1.03x10^-42^ |
| 9 | rs4977574 | *9p21* | 6.35x10^-98^ |
| 9 | rs579459 | *ABO* | 1.14x10^-10^ |
| 10 | rs2505083 | *KIAA1462* | 1.57x10^-10^ |
| 10 | rs2047009 | *CXCL12* | 2.75x10^-11^ |
| 10 | rs501120 | *CXCL12* | 1.39x10^-11^ |
| 10 | rs11203042 | *LIPA* | 1.22x10^-04^ |
| 10 | rs1412444 | *LIPA* | 5.15x10^-12^ |
| 10 | rs12413409 | *CYP17A1-CNNM2-NT5C2* | 1.07x10^-07^ |
| 11 | rs974819 | *PDGFD* | 2.44x10^-10^ |
| 11 | rs964184 | *ZNF259-APOA5-APOA1* | 5.60x10^-05^ |
| 12 | rs7136259 | *ATP2B1* | 2.45x10^-05^ |
| 12 | rs3184504 | *SH2B3* | 1.03x10^-09^ |
| 13 | rs9319428 | *FLT1* | 7.13x10^-05^ |
| 13 | rs4773144 | *COL4A1/A2* | 3.87x10^-07^ |
| 13 | rs9515203 | *COL4A1/A2* | 9.33x10^-10^ |
| 14 | rs2895811 | *HHIPL1* | 1.86x10^-05^ |
| 15 | rs7173743 | *ADAMTS7* | 5.55x10^-16^ |
| 15 | rs17514846 | *FURIN-FES* | 3.10x10^-07^ |
| 17 | rs216172 | *SMG6* | 5.07x10^-07^ |
| 17 | rs12936587 | *RAI1-PEMT-RASD1* | 8.24x10^-04^ |
| 17 | rs46522 | *UBE2Z* | 1.84x10^-05^ |
| 19 | rs1122608 | *LDLR* | 2.73x10^-11^ |
| 19 | rs2075650 | *APOE-APOC1* | 1.61x10^-06^ |
| 19 | rs445925 | *APOE-APOC1* | 4.23x10^-06^ |
| 21 | rs9982601 | *KCNE2* (gene desert) | 1.33x10^-13^ |

* The reported p-values refer to the meta-analysis work presented by Nikpay and colleagues (Nikpay et al., 2015).

** rs6903956 did not result significantly associated with CAD in the meta-analysis. The association p-value as calculated in the original paper is instead shown (Wang et al., 2011).

**Supplementary Table 4.** Results of the analysis performed on the SLE-associated loci (upper part) and on 1,000 random sets (lower part).

| **List** | ***n* blocks** | ***n* lncRNAs** | ***n* circRNAs** | ***n* miRNAs** | ***n* SEs** |
| --- | --- | --- | --- | --- | --- |
| SLE set | 34 | 18 | 197 | 6 | 14 |
|  |  |  |  |  |  |
| random sets* | 34.6 | 10.7 | 89.6 | 1 | 1.1 |
| SD | 2.7 | 3.8 | 46.4 | 1.4 | 1.2 |
| %** | - | 4.8 | 3.2 | 1.4 | 0 |
| *p-value* | - | 0.05 | 0.03 | 0.01 | 9.9*10^-4^ |

* For the random sets analysis, the average values calculated on 1,000 iterations are indicated.

*n*, number; SD, standard deviation.

** % of times in which the same or a larger number of lncRNAs, circRNAs, miRNAs, or SEs was obtained in the 1,000 iterations as compared to the SLE dataset.

P values were calculated as described (Davison and Hinkley, 1997); in detail: p value = (1+sum(s >= s0))/(N+1), where s is the observed value of the random set, s0 is the value of the observed SLE-specific result, and N is the number of bootstraps.

**Supplementary Table 5.** Results of the analysis performed on the RA-associated loci (upper part) and on 1,000 random sets (lower part).

| **List** | ***n* blocks** | ***n* lncRNAs** | ***n* circRNAs** | ***n* miRNAs** | ***n* SEs** |
| --- | --- | --- | --- | --- | --- |
| RA set | 58 | 28 | 145 | 0 | 9 |
|  |  |  |  |  |  |
| random sets* | 58.2 | 17.8 | 148.3 | 1.8 | 1.8 |
| SD | 3.6 | 4.9 | 59.5 | 2.1 | 1.5 |
| %** | - | 3.5 | 46.3 | 100 | 1.1 |
| *p-value* | - | 0.04 | 0.46 | 1.00 | 0.01 |

* For the random sets analysis, the average values calculated on 1,000 iterations are indicated.

*n*, number; SD, standard deviation.

** % of times in which the same or a larger number of lncRNAs, circRNAs, miRNAs, or SEs was obtained in the 1,000 iterations as compared to the RA dataset.

P values were calculated as described (Davison and Hinkley, 1997); in detail: p value = (1+sum(s >= s0))/(N+1), where s is the observed value of the random set, s0 is the value of the observed RA-specific result, and N is the number of bootstraps.

**
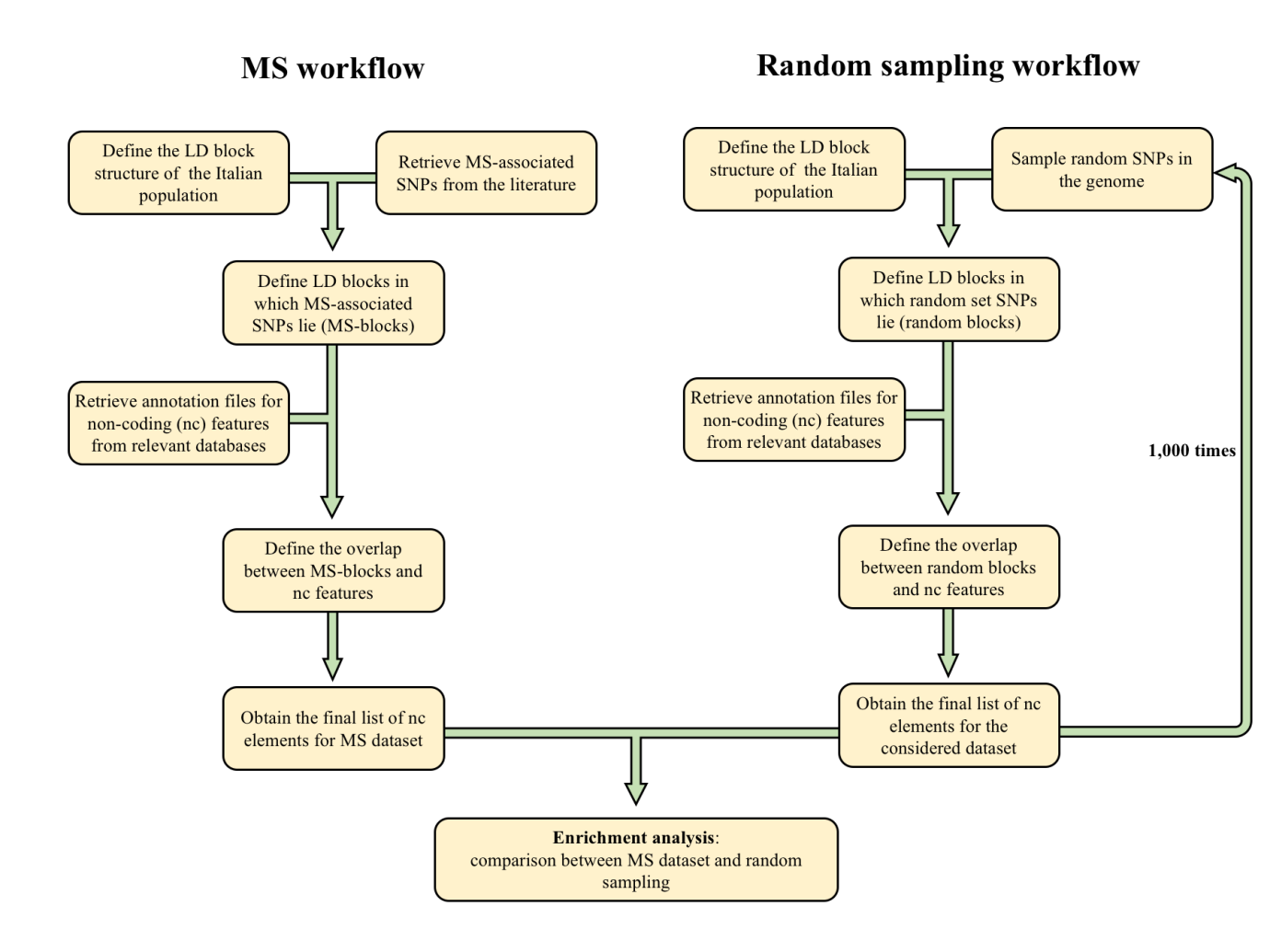
**

**Supplementary Figure 1. Flowchart representing the pipeline steps.**

**
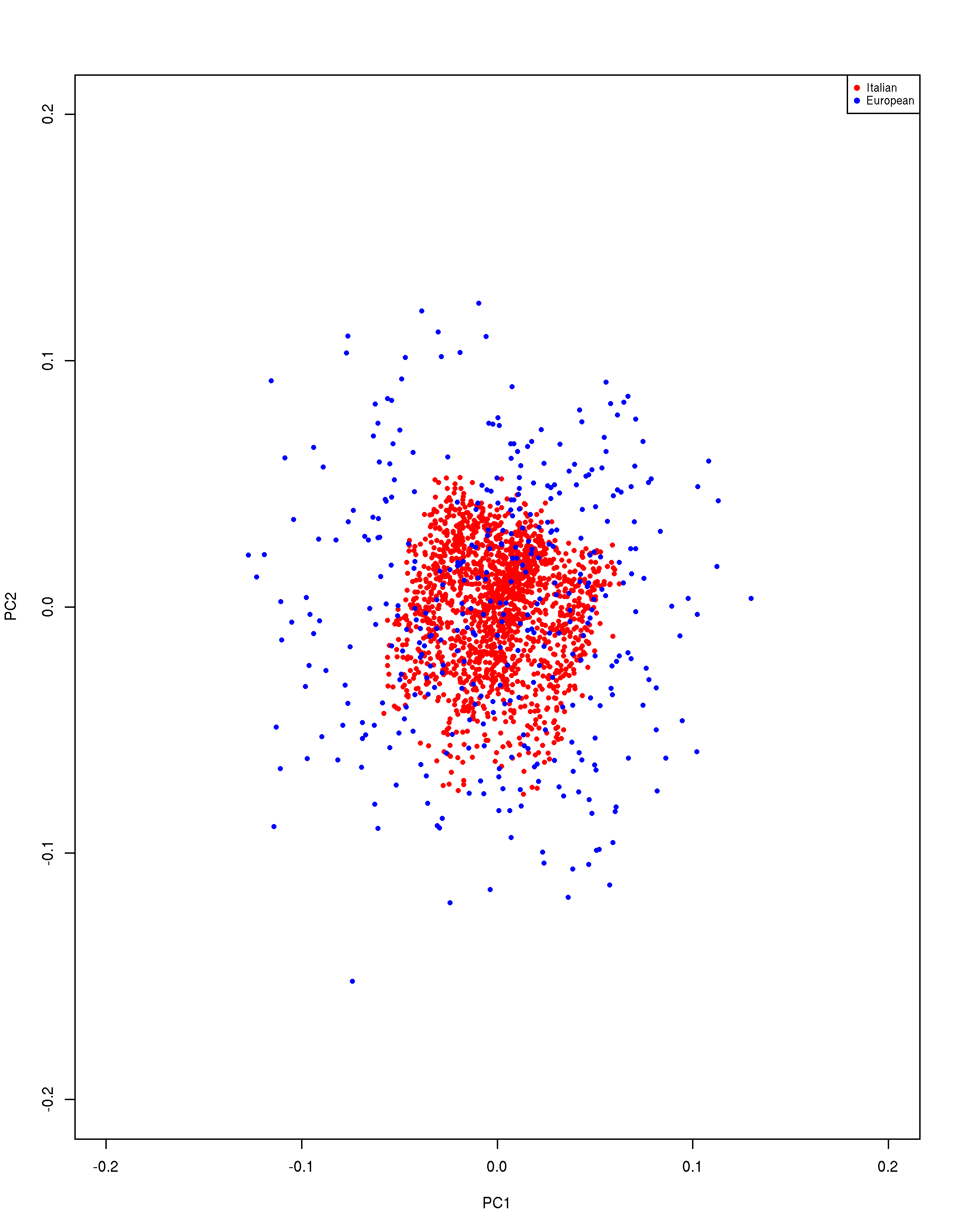
**

**Supplementary Figure 2. Principal components of ancestry in Italians (our cohort) and Europeans (1000 Genome project subjects).**

Principal component (PC) analysis of 1,668 healthy unrelated individuals of self-reported Italian origin (red dots) and of 503 individuals of European origin (from the 1000 Genome project, phase 3; blue dots). The two PCs are shown (PC1 and PC2), demonstrating a substantial overlap between the two populations. PC analysis was preformed using SmartPCA software contained in the EIGENSOFT package (Patterson et al., 2006; Price et al., 2006).

**
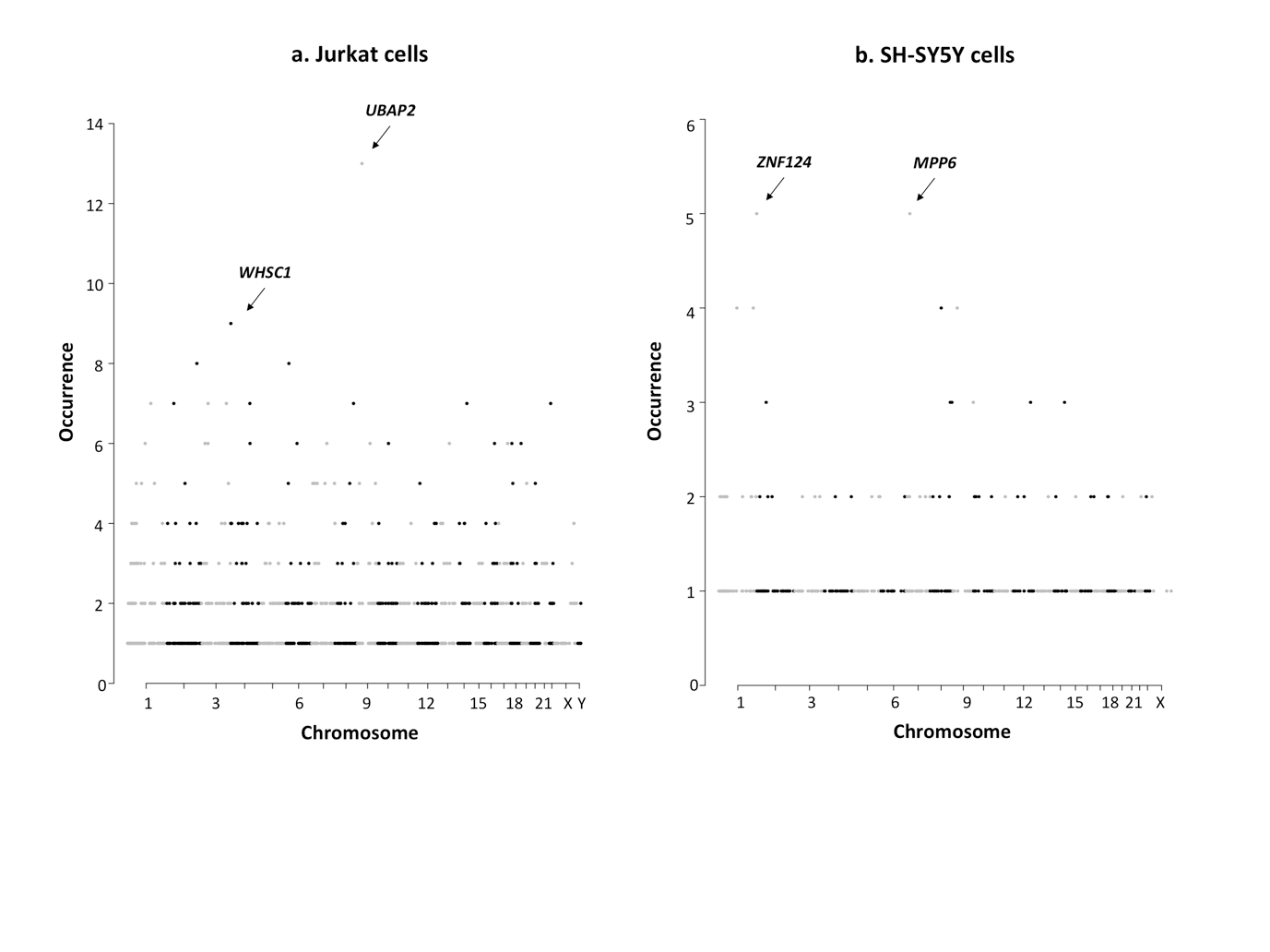
**

**Supplementary Figure 3. Distribution of circRNAs per gene.**

The figure shows the number of different species of circRNAs (supported by at least 5 reads) carried by each gene for Jurkat cells (panel a) and SH-SY5Y cells (panel b). Genes are separated according to their position on chromosomes (X axis) and represented as dots. The number of different circRNAs is reported on Y axis. Arrows indicate the genes characterized by the highest number of circRNAs, and their names are also shown.

**References**

Davison, A.C., Hinkley, D.V. (1997). Bootstrap methods and their application. New York: Cambridge University Press

International Multiple Sclerosis Genetics Consortium (IMSGC), Beecham, A.H., Patsopoulos, N.A., Xifara, D.K., Davis, M.F., Kemppinen, A., et al. (2013). Analysis of immune-related loci identifies 48 new susceptibility variants for multiple sclerosis. *Nat Genet*. 45, 1353-1360. doi: 10.1038/ng.2770

Nikpay, M., Goel, A., Won, H.H., Hall, L.M., Willenborg C., Kanoni S., et al. (2015). A comprehensive 1,000 Genomes-based genome-wide association meta-analysis of coronary artery disease. *Nat Genet*. 47, 1121-1130. doi: 10.1038/ng.3396

Patterson, N., Price, A.L. and Reich, D. (2006) Population structure and eigenanalysis. *PLoS Genet*., 2, e190. doi: 10.1371/journal.pgen.0020190

Price, A.L., Patterson, N.J., Plenge, R.M., Weinblatt, M.E., Shadick, N.A. and Reich, D. (2006). Principal components analysis corrects for stratification in genome-wide associa- tion studies. *Nat. Genet*., 38, 904-909. doi: 10.1038/ng1847

Wang, F., Xu, C.Q., He, Q., Cai, J.P., Li, X.C., Wang, D., et al. (2011). Genome-wide association identifies a susceptibility locus for coronary artery disease in the Chinese Han population. *Nat Genet*. 43, 345-349. doi: 10.1038/ng.783
